# Balance deficits in long-term pediatric ALL survivors

**DOI:** 10.18632/oncotarget.25967

**Published:** 2018-08-24

**Authors:** Mitra Varedi, Kirsten K. Ness, Raymond F. McKenna

**Affiliations:** Health and Rehabilitation Sciences Program, Stony Brook University, Stony Brook, NY, USA

**Keywords:** acute lymphoblastic leukemia, late effects, cognitive dysfunction, postural balance

A person’s ability to maintain balance emerges from a complex interaction among sensory systems that makes that person aware of his/her location in regards to the environment, higher-level central nervous system processes that analyze and interpret sensory information and plan movement strategies, and the motor system that executes these strategies [1]. The treatment for acute lymphoblastic leukemia (ALL) can impair all of these body systems resulting in difficulties with movement and balance [2]. As adequate balance is a prerequisite for stabilizing the body in upright positions during voluntary movements, deficits in balance may help explain why childhood ALL survivors have lower physical activity levels and lower participation rates in sports [3, 4].

In a recent study of long-term childhood ALL survivors [5], we examined the potential effect of peripheral neuropathy on static and dynamic measures of balance. As hypothesized, the level of peripheral neuropathy was associated with poorer performance on dynamic measures of mobility (i.e., Timed Up and Go test) and endurance (i.e., 6-Minute Walk test); however the level of peripheral neuropathy was not associated with sway values for maintaining an upright posture on a dynamic posturography device. Another examined factor, visual-motor processing speed, which was obtained from the Digit-Symbol Coding test from the Wechsler Adult Intelligence Scale–Fourth Edition, was associated with balance performance on all investigated measures. Survivors with slower visual-motor processing speed had more sway on the computerized posturography device, took longer to finish the Timed Up and Go test, and achieved a shorter distance in the 6-Minute Walk test. The associations between visual-motor processing speed and static and dynamic measures of balance suggest that cognitive deficits may be a key factor in the observed balance deficits among ALL survivors. Based on these findings, we suggest the integration of cognitive activities when assessing balance in ALL survivors.

Outside of the clinic, balance tasks are rarely performed in isolation. Often, maintaining balance is combined with simultaneously performing a cognitive activity, such as walking and attending to a conversation. The performance of two tasks is unaffected if the person has enough attentional resources (i.e., the information-processing resources that are required to complete a task [1]); otherwise, performance on the cognitive task, balance task, or both can be impaired. Many long-term ALL survivors experience deficits in muscle strength and flexibility [6]. To compensate for this physical disadvantage, they may devote more attentional resources to perform a dynamic task, such as walking on a busy street. However, survivors may also have limited attentional resources, compared to their healthy peers. The negative effects of anticancer treatment on cognitive function in ALL survivors have been well documented; these deficits include structural alterations in the brain, decreased processing speed, and attention deficits [7, 8]. Given these limitations in physical and cognitive performance, long-term ALL survivors may be more challenged to maintain their upright posture during movement and particularly in situations that involve additional attentional demands, such as walking on a busy street and attending to a conversation. Therefore, evaluating balance while a survivor performs a cognitive task (i.e., a cognitive-motor paradigm) is an appropriate strategy to examine his/her ability to maintain an upright posture as this setting more closely resembles the functional requirements of everyday life.

These findings emphasize the application of cognitive–motor task training to address balance deficits and gait limitations among long-term childhood ALL survivors. By utilizing rehabilitation approaches that concurrently challenge survivors’ physical and cognitive functions, rather than those that individually target balance and gait, survivors may be able to better transfer their improvements to real-life situations.
